# Impact of incisional hernia on quality of life after colorectal cancer surgery – results of the Rein4CeTo1 randomized clinical trial

**DOI:** 10.3389/jaws.2026.17039

**Published:** 2026-08-11

**Authors:** Charlotta L. Wenzelberg, Carl-Fredrik Rönnow, Ulf Petersson, Peder Rogmark

**Affiliations:** 1 Department of Clinical Sciences Malmö, Lund University, Lund, Sweden; 2 Department of Surgery, Skånes University Hospital Malmö, Malmö, Sweden

**Keywords:** colorectal cancer (CRC), incisional hernia (IH), malignant disease, quality of life (QoL), stoma

## Abstract

**Purpose:**

To evaluate quality of life (QoL), abdominal wall pain and discomfort 1 and 3 years after colorectal cancer (CRC) surgery in patients with and without incisional hernia (IH) and perform a risk factor analysis for impaired QoL.

**Methods:**

Longitudinal observational study with patients included in the randomized controlled trial, Rein4CeTo1, carried out 2017–2021. Patients underwent CT-scans 12 ± 3 and 36 ± 3 months after surgery, scrutinized for diagnosis of IH. General health was evaluated using the EQ-5D-5L questionnaire. For QoL related to IH and overall abdominal wall symptoms a slightly modified version of the Ventral Hernia Pain Questionnaire (VHPQ) was used. Potential risk factors of impaired QoL were investigated with multivariable linear regression analysis.

**Results:**

Of 160 randomized patients, 134 were eligible for 1-year follow-up and 119/134 (89%) answered the questionnaires. Corresponding numbers at 3 years was 81/101 (80%). The EQ-5D-5L results were similar at 1 and 3 years for patients with and without IH (p = 0.276 1 year, p = 0.404 3 years). The VHPQ concluded low frequency of pain, stiffness in the abdominal wall and other discomforting symptoms without differences between patients with and without IH. In multivariable linear regression stoma was an independent risk factor for impaired general QoL at 3 years, p < 0.005.

**Conclusion:**

In this study, comparing QoL 1 and 3 years after CRC surgery, we could not find a statistically significant difference in patients developing an IH compared to those who did not. The presence of a stoma was an independent risk factor for impaired general QoL after 3 years.

## Introduction

Patients’ health perception and satisfaction are utmost important outcomes after any medical treatment and many instruments for evaluation of patient reported outcome measures (PROM) and patient reported experience measures have been developed. Generic instruments assess overall health perception while disease specific instruments measure outcomes for a particular disease or treatment. The World Health Organization (WHO) defines Quality of Life (QoL) as “an individual’s perception of their position in life in the context of the culture and value systems in which they live and in relation to their goals, expectations, standards and concerns” [1] and the Centers for Disease Control and prevention describes health-related quality of life as “an individual’s or a group’s perceived physical and mental health over time” [2].

Incisional hernia (IH) is a common long-term complication to abdominal surgery with negative impact on QoL [3], increased surgical morbidity and societal costs [4]. Several instruments designed to evaluate QoL in patients with IH are available [5]. In the absence of consensus on the most appropriate, many different instruments are used which complicates comparison of QoL between studies. Furthermore, the length of follow-up varies greatly in the literature, aggravating comparisons even more [6]. In a systematic review from 2021, Grove et al. stated that the use of QoL assessment tools, both generic and disease-specific, is gold standard in surgery in general, and that a single accepted tool for assessing QoL in abdominal wall hernia patients is still lacking [5].

Patients with colorectal cancer (CRC) exhibit impaired QoL compared to the population norm [7], and CRC patients are also prone to develop IH [8]. What impact IH has on QoL in CRC patients is sparsely investigated but a previous Danish register-study indicated an additional decrease in QoL in CRC patients developing an IH [9].

In the prospective randomized controlled trial (RCT) Rein4CeTo1, on patients undergoing elective surgery for CRC, a Reinforced Tension Line (RTL) suture [10] was combined with the small bite 4:1 closure technique [11] which resulted in a lower incidence of CT-diagnosed IH when compared to standard small bite 4:1 closure alone [12, 13]. It has been shown before that the RTL fascial closure with RTL plus 4:1 decreases incisional hernia incidence [14].

The main aim of this study was to evaluate QoL, abdominal wall pain and discomfort 1 and 3 years after CRC surgery in patients included in the Rein4CeTo1 study, and to compare these outcomes in patients with and without an incisional hernia, using one generic and one disease specific QoL assessment questionnaire.

## Materials and methods

### Study design

The Rein4CeTo1 randomized controlled trial [12] was carried out at Skåne university hospital in Malmö and the County hospital Kristianstad in Sweden between 2017 and 2021. The primary aim of the Rein4CeTo1 trial was to investigate CT-diagnosed IH incidence at 1 year, comparing fascia closure with an RTL-suture combined with the small bite 4:1 suturing technique, using polypropylene sutures (the RTL-group), to standard small bite 4:1 closure alone, using polydioxanone sutures (the PDS-group), after CRC surgery through a midline incision. Patients scheduled for open CRC surgery were eligible for inclusion. Exclusion criteria were age under 18 years; ASA score higher than III; earlier midline hernia surgery or current midline hernia over 1 cm; planned CRS/HIPEC operation or peritoneal carcinomatosis; patients perioperatively assessed by the surgeon as not suitable or needing fascia reconstruction; and patients presumably unable to participate in follow-up. Early and long-term results from the study were recently published [12, 13].

The objectives for this study were to measure patients’ global quality of life and symptoms related to the abdominal wall at 1 year and 3 years follow-ups using two questionnaires, comparing patients with and without IH. In addition, a risk factor analysis, with predefined, known risk factors for impaired QoL including IH, stoma, CRC recurrence or new malignant disease, was performed.

Included in this longitudinal observational study were Rein4CeTo1-patients answering the questionnaires and undergoing CT-scans 12 ± 3 and 36 ± 3 months after surgery that were used for IH diagnosis. Reoperated patients were excluded from analysis.

The Rein4CeTo1 trial was approved by The Regional Ethics Committee at Lund University, Sweden (Dnr 2017/459) and registered at www.clinicaltrials.gov (NCT03390764).

### Clinical examination and questionnaires

The patients were invited to an out-patient visit, 1 and 3 years after surgery for a clinical examination focusing on IH diagnosis and were at the same time asked to fill out two questionnaires.

General health related QoL was evaluated using the EQ-5D-5L questionnaire developed by the EuroQol Group [15]. The EQ-5D-5L descriptive system consists of five dimensions: mobility (MO), self-care (SC), usual activities (UA), pain/discomfort (PD), and anxiety/depression (AD). Each dimension has five levels: 1 = no problems, 2 = slight problems, 3 = moderate problems, 4 = severe problems, and 5 = unable/extreme problems. A patient’s unique health state or “health profile” is defined by combining the number of the severity level from each of the 5 dimensions. The possible health states range from 1-1-1-1-1 at the best to 5-5-5-5-5 at the worst, resulting in one of 3125 possible health states. Each level of the dimensions is assigned a weight, reflecting the impact on QoL. An EQ-5D index between 1 (best) and 0 (worst) can then be computed by subtracting the dimension weight values from 1. A Swedish value set for the descriptive system is available [16]. In addition, the EQ-5D-5L includes a visual analogue scale for self-rating of overall health status, EQ-VAS, where patients rate their health status ranging from 0 (‘worst health you can imagine’) to 100 (‘best health you can imagine’). The EQ VAS can be used as a quantitative measure of health [17]. The use of EQ-5D-5L was approved for research use by the EuroQol group, ID:21776.

For QoL specifically related to IH a slightly modified version of the disease specific Ventral Hernia Pain Questionnaire (VHPQ) [18] was used, approved by the inventors. The VHPQ is a questionnaire for assessment of pain and its impact on activity and function, related to ventral hernia and ventral hernia repair, consisting of 19 questions. The first six questions of the VHPQ concern the intensity, frequency and duration of pain. The next seven questions relate to the impact on daily activities and need for medication. The final six questions deal with work limitation, subsequent operations, patient satisfaction and how physically demanding the patients regard their occupation. The validity and reliability of the VHPQ makes it a useful tool in assessing preoperative and postoperative pain and patient satisfaction. Two questions relate to pain intensity and the patients are asked to describe their pain “right now” and worst pain “last week” according to a 7-grade scale: 1 = no pain; 2 = pain that can easily be ignored; 3 = pain that cannot be ignored but does not affect daily activities; 4 = pain that cannot be ignored which affects concentration and daily activities; 5 = pain that inhibits most daily activities; 6 = pain that requires rest or bed rest; and 7 = pain so severe that you are forced to seek medical attention. Ignorable pain was defined as grade 1 and 2 and non-ignorable pain was defined as grade 3 or worse.

In addition to the EQ-5D-5L and the VHPQ, included patients were asked questions concerning satisfaction with cosmesis and overall outcome of the operation. Patients were asked, and medical records searched, for information on recurrence of CRC or other newly diagnosed malignant disease, and whether subsequent abdominal surgery had been performed.

### Data analysis and statistics

QoL and abdominal wall pain and discomfort were secondary aims in the Rein4CeTo1 RCT and no power calculation was performed for these outcomes. Continuous variables are presented as mean with standard deviation (SD) and evaluated with Student’s t-test. Categorical variables were analysed with Pearson Chi^2^-test or Fisher exact test, if the number for any expected outcome was below 5. All tests were 2-sided. The EQ-5D-5L health states and EQ VAS were compared with the Swedish characteristics developed by Burstrom et al. [16]. An EQ-5D_index_, was calculated using the value set of the time trade-off (TTO) model [16]. A 95% confidence interval (CI) is reported. The EQ-5D_index_ was used in multivariable linear regression analysis for evaluation of possible risk factors.

A *p*-value of ≤0.05 was considered statistically significant. All statistical analyses were performed using IBM SPSS Statistics version 26.0.0.1 software.

## Results

A total of 160 patients were randomized in the Rein4CeTo1 trial, whereof 134 were eligible for 1-year follow-up and 119/134 patients (89%) answered the questionnaires 1 year after surgery. Of the 101 remaining patients at 3 years, 81 (80%) patients filled out the questionnaires. In total, 78 patients answered the questionnaires on both occasions.

### EQ-5D-5L

The EQ-5D-5L descriptive results of the present cohort of patients did not deviate from the Swedish characteristics, except for a possibly larger portion of patients scoring a lower level in the usual activity and the pain/discomfort dimensions, see Table 1. The results at 1 and 3 years were similar for patients with and without IH, respectively.

**TABLE 1:** The EQ-5D-5L results (mean, confidence interval (CI)) of the dimensions usual activity and pain/discomfort, and the Swedish characteristics.

EQ-5D-5L dimension	Total 1Y (n = 119)	1Y no IH (n = 101)	1Y IH (n = 18)	Total 3Y (n = 81)	3Y no IH (n = 63)	3Y IH (n = 18)	Swedish characteristics^1^
Usual activity 1 (95% CI)	78.2 (70.1–84.8)	77.2 (68.4–84.6)	83.3 (61.9–95.1)	76.5 (66.5–84.7)	81.0 (70.0–89.1)	61.1 (38.3–80.6)	69.2 (68.6–69.8)
2 (95% CI)	109 (6.3–17.5)	12.9 (7.4–29.4)	0.0	13.6 (7.4–22.3)	9.5 (4.1–18.6)	27.8 (11.5–50.6)	18.2 (17.7–18.6)
3–5 (95% CI)	10.9 (6.3–17.5)	9.9 (5.2–16.9)	16.7 (4.9–38.1)	9.9 (4.8–17.8)	9.5 (4.1–18.6)	11.1 (2.4–31.1)	12.6 (12.2–13.0)
Pain/Discomfort 1 (95% CI)	71.4 (62.9–79.0)	70.3 (60.9–78.5)	77.8 (55.4–92.0)	51.9 (41.1–62.5)	52.4 (40.2–64.4)	50.0 (28.4–71.6)	31.9 (31.4–32.5)
2 (95% CI)	14.3 (8.9–21.4)	15.8 (9.7–23.9)	5.6 (0.6–23.1)	30.9 (21.6–41.5)	28.6 (18.6–40.5)	38.9 (19.4–61.7)	39.9 (39.3–40.4)
3–5 (95% CI)	14.3 (8.9–21.4)	13.9 (8.2–21.6)	16.7 (4.9–38.1)	17.3 (10.3–26.6)	19.0 (10.9–30.0)	11.1 (2.4–31.1)	28.2 (27.7–28.8)

CI: Confidence Interval.

1 Burström et al [16].

All dimensions levels are: 1 = no problems, 2 = slight problems, 3 = moderate problems, 4 = severe problems, 5 = unable/extreme problems.

The EQ-5D-5L results for all 5 dimensions (mobility, self-care, usual activities, pain/discomfort and anxiety/depression) at 1 compared to 3 years were without significant differences and are shown in Supplementary Table 1.

**FIGURE 1:**
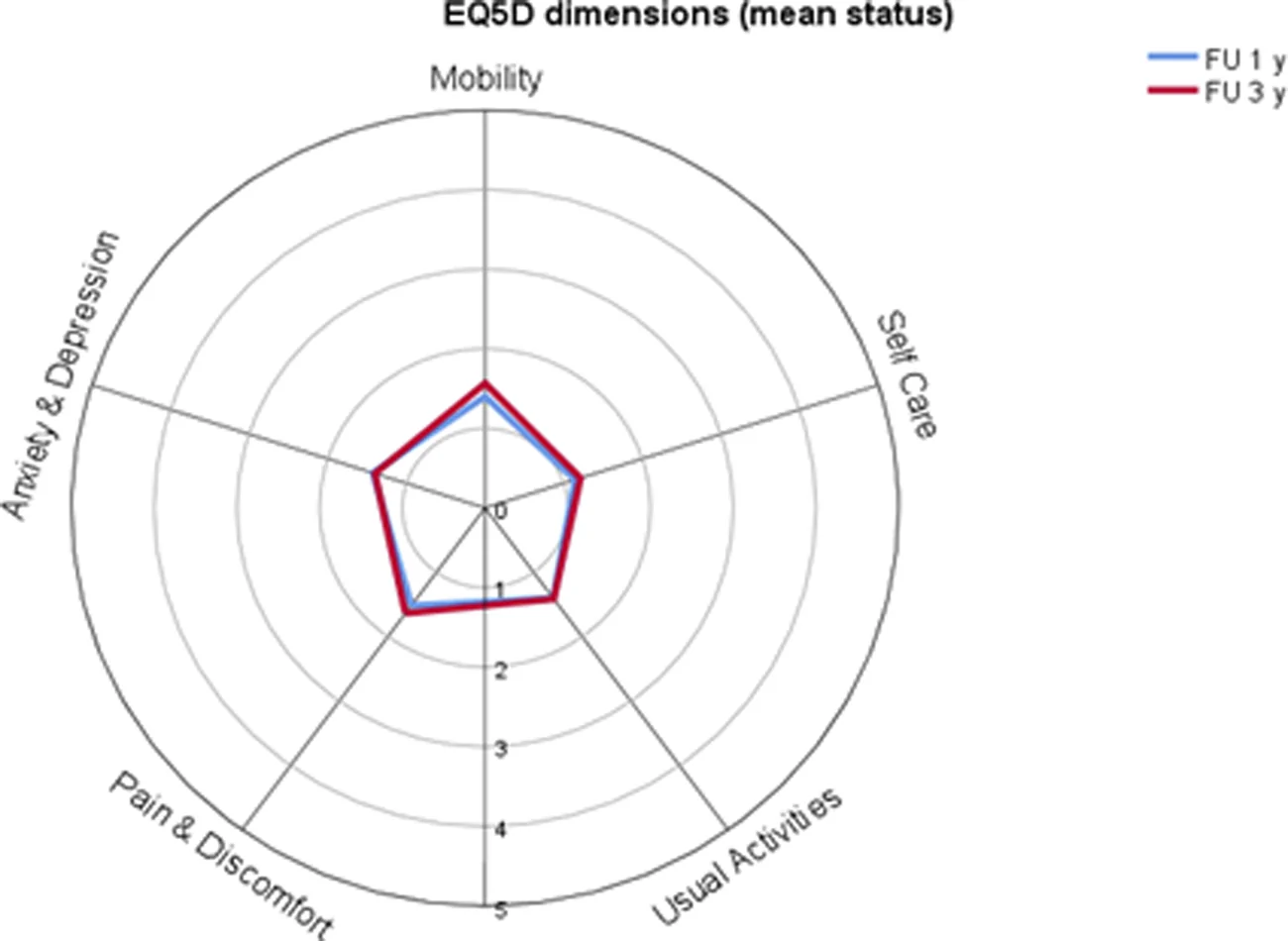
Mean values of the different dimensions at 1 and 3 years. Scale levels 1 (best)…5 (worst). N = 119 at 1 year and N = 81 at 3 years.

Mean EQ-5D-5L values for each dimension were all ≤1.5 at 1 year and ≤1.7 at 3 years as shown in Figure 1, corresponding to in between ‘no’ and ‘slight’ problems.

**FIGURE 2:**
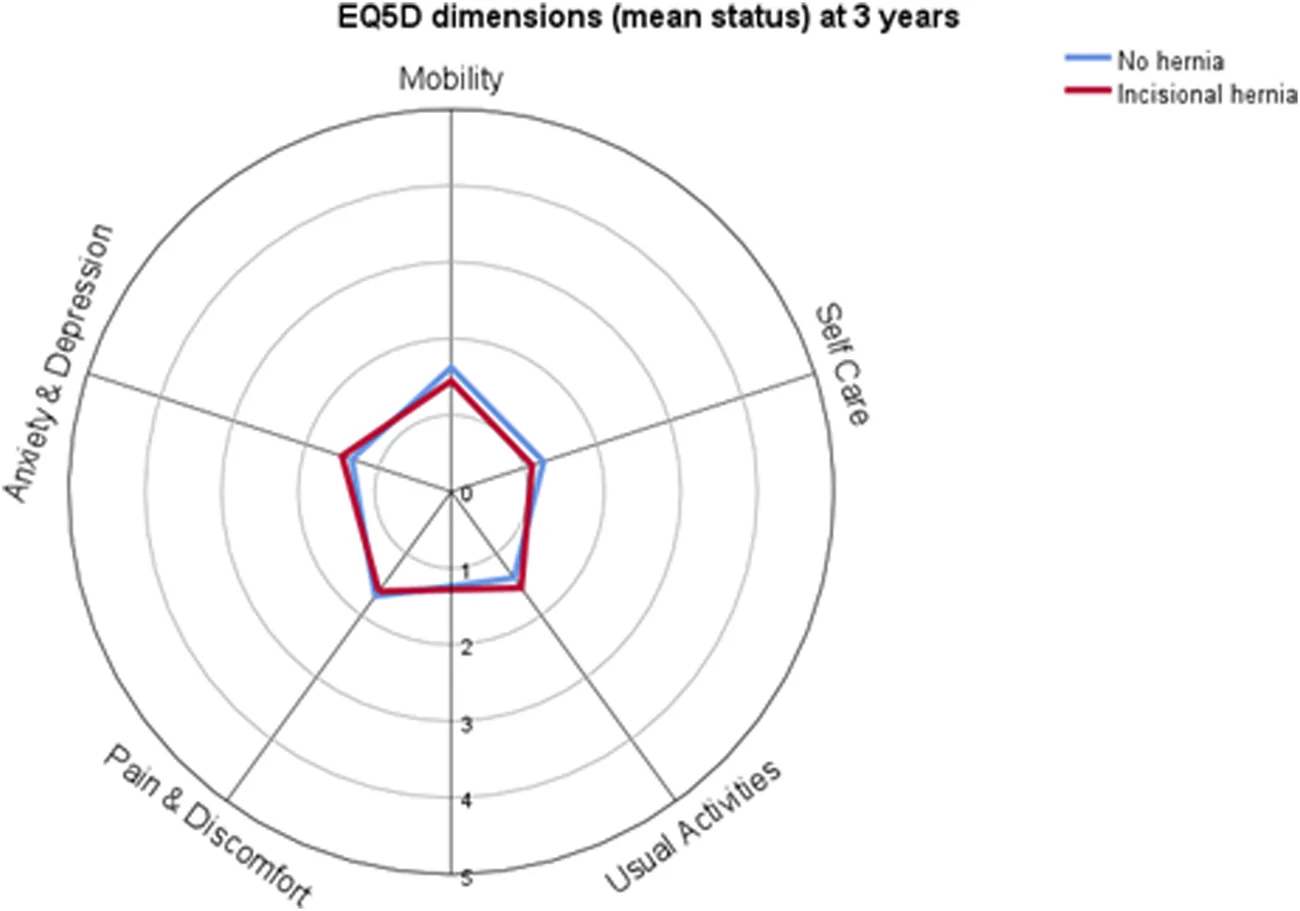
Mean values of the different dimensions for patients with and without IH at 3 years. Scale levels 1 (best)…5 (worst). N = 63 for no hernia and N = 18 for hernia.

Patients with IH at 1 year or 3 years follow-up did not report worse overall QoL, compared to those without IH (*p* = 0.276 at 1 year and *p* = 0.404 at 3 years). Mean EQ-5D-5L values for each dimension in patients with and without IH, respectively, at 3 years are presented in Figure 2. Despite excluding IH < 2 cm (fascial gaps without extrusion, *n* = 3), which was presumed less clinically significant, the remaining larger IH were not found to be an independent risk factor for impaired QoL.

### EQ-VAS

The mean EQ-VAS at 1 compared to 3 years, were without significant differences and did not deviate from the Swedish characteristics, see Table 2.

**TABLE 2:** The EQ VAS results and the Swedish characteristics.

EQ VAS	Total 1Y (n = 119)	1Y no IH (n = 101)	1Y IH (n = 18)	Total 3Y (n = 81)	3Y no IH (n = 63)	3Y IH (n = 18)	Swedish characteristics^1^
Mean (SD)	78 (15.6)	79 (14.5)	75 (18.6)	77 (17.0)	77 (17.1)	77 (17.4)	76.1 (18.7)

1 Burström et al [16].

### Risk factors of impaired QoL

In the multivariable linear regression IH, recurrence of CRC or new malignant disease, and stoma were included as potential risk factors for impaired QoL. None was identified as an independent risk factor at 1 year. At 3 years the presence of a stoma was found to be an independent risk factor for impaired general QoL, *p* = 0.004, see Table 3.

**TABLE 3:** Risk factor analysis for QoL (linear regression coefficients).

​	1 year 119 patients				3 years 78 patients			
Risk factor	N	B	95% CI	p-value	N	B	95% CI	p-value
Incisional hernia	18 (15%)	−0.027	−0.075-0.022	0.276	18 (23%)	−0.024	−0.080, 0.032	0.404
Recurrent CRC/New malignant disease	9 (8%)	0.015	−0.050-0.080	0.656	8 (10%)	−0.058	−0.136, 0.020	0.142
Stoma present	68 (57%)	−0.004	−0.039-0.030	0.803	40 (51%)	−0.070	−0.117, −0.022	0.004*

N = number of patients, B = regression coefficient, 95% CI = 95% confidence interval. * = p-value < 0.05.

### VHPQ

Non-ignorable pain “right now” at 1 year was reported by 5.0%, and at 3 years by 1.2%. The corresponding figures for non-ignorable pain “last week” were 3.4% and 2.5%, respectively.

Sensation of stiffness in the abdominal wall was present in 11.0% at 1 year, and 7.4% at 3 years, and other discomforting symptoms was reported by 11.9% at 1 year, and 6.3% at 3 years. There were overall low frequency (occasional or several times/week) and short duration of pain (minutes to hours) with low impact on daily activities and hardly any need for analgesic medication at both follow-up occasions. No patient at the time of the follow-up was on sick leave due to pain. In this cohort 55% were retired at 1 year and 70% at 3 years.

In all, 23.5% of patients regarded their scar as cosmetically disturbing at 1 year and 19.8% at 3 years, and 10.9% of patients experienced the appearance of their abdominal wall as somehow socially limiting at 1 year, and 10.0% at 3 years. Still, the patients were content with the result of their operation in 87.1% at 1 year, and 92.5% at 3 years.

There were no differences at any of the follow-up occasions between the RTL group and the PDS group, nor were there any differences between patients with IH compared to those without.

## Discussion

In this study, patients from the Rein4CTo1 trial were evaluated regarding general health related QoL and abdominal wall pain and discomfort at 1 and 3 years after CRC surgery, hypothesizing that patients developing an IH would report worse outcome. We found no statistically significant differences, neither in general QoL nor in pain and discomfort related to the abdominal wall, between patients with and without IH. The results of general QoL at 1 and 3 years postoperatively were in accordance with the Swedish characteristics. Risk factor analysis concluded that the presence of a stoma was an independent risk factor for impaired general QoL at 3 years.

The patients with IH did not have a lower general QoL compared to the patients without IH and the patients in this study reported mean scores in the different dimensions corresponding to ‘no’ to ‘slight’ problems, and a proportional distribution between the levels in the different dimensions and mean EQ-VAS did not differ from the Swedish characteristics. These findings are contradictory to some previous studies showing that IH have a negative impact on QoL [19]. Rogmark et al. reported improved and normalized QoL after IH repair compared to preoperative assessment using SF-36 in an RCT comparing laparoscopic to open IH repair. In addition, two other studies using SF-36 found reduced physical function in patients with IH, but no reduction in general health [20, 21], possibly due to lack of statistical power. Results reported by Jensen et al. in a Danish register-study on CRC patients developing an IH, indicate an additional impairment in QoL based on the disease specific questionnaire EORTC QLQ-C30 [9]. Additionally, Toma et al. demonstrated a significant overall improvement of QoL measured with EQ-5D-5L questionnaire after abdominal wall reconstruction [17], altogether indicating that IH has a negative impact on QoL even if the use of several different QoL instruments complicates evaluation and comparison between studies [5]. A possible reason to our results, is that general QoL have a broad meaning, covering much more than symptoms from the abdominal wall, and the CRC patient cohort in this study present several possible factors affecting general QoL. The absence or presence of an IH may not have a dominant impact in this respect. Furthermore, sensitivity for different QoL instruments may vary when used in cohorts with diverse complexity.

Swedish CRC patients have previously been shown to report an impaired QoL compared to the population norm [7]. In a systematic review from 2021, it was found that the average health related QoL score of patients with CRC in the palliative phase was lower than for the general population, but comparable to that of long-term survivors of CRC from previous studies [22]. Also, in this respect our results are contradictory with QoL at the same level as the overall Swedish population. The relatively small number of patients in this study compared to the above-mentioned reports make our results relatively precarious.

In this cohort with relatively few IHs, the number of risk factors possible to include in the regression analysis was limited, and the previously described risk factors IH, CRC/new malignant disease and stoma were predefined. We found that having a stoma 3 years after CRC surgery was an independent risk factor for general impaired QoL, in accordance with previous studies. A systematic review of ostomy-related problems and their impact on QoL of colorectal cancer ostomates concluded that living with a colostomy influences the overall QoL negatively [23].

The abdominal wall and IH specific questionnaire, VHPQ, showed low incidence and frequency of pain, stiffness in the abdominal wall and other discomforting symptoms without any difference comparing patients with and without IH. Furthermore, there was no difference between the RTL and the PDS groups. Most of the patients were content with the result of their operation, despite one forth regarding their scar as cosmetically disturbing and some patients even experiencing their abdominal wall as socially limiting.

Our study is strengthened by the prospective design, including patients participating in an RCT with standardized inclusion criteria and two recruiting centres. However, our study contains certain limitations. Firstly, one possible explanation to the contradictory result in the present study could be that the general QoL and specific questions regarding the abdominal wall were secondary aims of the Rein4CeTo1 RCT and no power calculation was performed for these outcomes, inflicting a risk of the study being under-powered.

Secondly, our study is further limited by a significant portion of included patients being lost to follow-up, in part due to travelling restrictions during the pandemic.

Lastly, our study is also limited by absence of preoperative questionnaire data preventing us from evaluating possible changes in QoL preoperatively and postoperatively. In the current study we have followed the patients over a 3-year period after the CRC operation and found similar general QoL results over time. One reason for refraining from a preoperative questionnaire was that patients were asked to participate and included in the Rein4CeTo1 trial the day before their CRC surgery, where preoperative anxiety was believed to distort the results of a questionnaire for assessment of general QoL. Furthermore, the aim of this part of the Rein4CeTo1 trial was to evaluate the impact on QoL of IH development postoperatively, why a preoperative questionnaire survey was not of primary interest, as stated above.

## Conclusion

QoL in patients developing an IH after CRC surgery is sparsely investigated, and a single widely accepted tool for QoL-assessment is lacking. In this study, comparing QoL 1 and 3 years after CRC surgery in patients participating in the RCT Rein4CeTo1, we could not find a statistically significant difference in general QoL or abdominal wall symptoms for patients developing an IH compared to those who did not. The presence of a stoma was found to be an independent risk factor for impaired general QoL after 3 years. The results need to be interpreted with caution since there are weaknesses in this study. Further studies are warranted were QoL assessment constitutes the primary aim.

## Data Availability

The raw data supporting the conclusions of this article will be made available by the authors, without undue reservation.
