# Supplementary material for: Impact of incisional hernia on quality of life after colorectal cancer surgery – results of the Rein4CeTo1 randomized clinical trial
Source: J Abdom Wall Surg. 2026 Aug 11;5:17039. doi: 10.3389/jaws.2026.17039 (PMC13503296; doi:10.3389/jaws.2026.17039)
Supplement: Supplementary file 1 [file Table1.docx]

| EQ5D-5L  dimension | Total 1Y  (n = 119) | 1Y no IH  (n = 101) | 1Y IH  (n = 18) | Total 3Y  (n = 81) | 3Y no IH  (n = 63) | 3Y IH  (n = 18) | Swedish characteristics^1^ |
| --- | --- | --- | --- | --- | --- | --- | --- |
| Mobility  1  (95% CI) | 78.2  (70.1-84.8) | 78.2  (69.4-85.4) | 77.8  (55.4-92.0) | 65.4  (54.7-75.1) | 66.7  (54.5-77.4) | 61.1  (38.3-80.6) | 67.6  (67.0-68.2) |
| 2  (95% CI) | 10.9  (6.3-17.5) | 12.9  (7.4-20.4) | 0.0 | 21.0  (13.2-30.8) | 17.5  (9.6-28.2) | 33.3  (15.3-56.3) | 17.2  (16.7-17.7) |
| 3-5  (95% CI) | 10.9  (6.3-17.5) | 8.9  (4.5-15.6) | 22.2  (8.0-44.6) | 13.6  (7.4-22.3) | 15.9  (8.5-26.3) | 5.6  (0.6-23.2) | 15.2  (14.8-15.6) |
| Self-Care  1  (95% CI) | 92.4  (86.7-96.2) | 94.1  (88.2-97.5) | 83.3  (61.9-95.1) | 90.1  (82.2-95.2) | 88.9  (79.4-94.9) | 94.4  (76.8-99.4) | 89.9  (88.8-89.6) |
| 2  (95% CI) | 5.0  (2.1-10.1) | 4.0  (1.4-9.1) | 11.1  (2.4-31.1) | 3.7  (1.1-9.6) | 4.8  (1.4-12.2) | 0.0 | 6.0  (5.7-6.3) |
| 3-5  (95% CI) | 2.5  (0.7-6.6) | 12.0  (0.4-6.2) | 5.6  (0.6-23.2) | 6.2  (2.4-13.0) | 6.3  (2.2-14.4) | 5.6  (0.6-23-2) | 4.8  (4.5-5.1) |
| Usual Activities  1  (95% CI) | 78.2  (70.1-84.8) | 77.2  (68.4-84.6) | 83.3  (61.9-95.1) | 76.5  (66.5-84.7) | 81.0  (70.0-89.1) | 61.1  (38.3-80.6) | 69.2  (68.6-69.8) |
| 2  (95% CI) | 10.9  (6.3-17.5) | 12.9  (7.4-29.4) | 0.0 | 13.6  (7.4-22.3) | 9.5  (4.1-18.6) | 27.8  (11.5-50.6) | 18.2  (17.7-18.6) |
| 3-5  (95% CI) | 10.9  (6.3-17.5) | 9.9  (5.2-16.9) | 16.7  (4.9-38.1) | 9.9  (4.8-17.8) | 9.5  (4.1-18.6) | 11.1  (2.4-31.1) | 12.6  (12.2-13.0) |
| Pain/Discomfort  1  (95% CI) | 71.4  (62.9-79.0) | 70.3  (60.9-78.5) | 77.8  (55.4-92.0) | 51.9  (41.1-62.5) | 52.4  (40.2-64.4) | 50.0  (28.4-71.6) | 31.9  (31.4-32.5) |
| 2  (95% CI) | 14.3  (8.9-21.4) | 15.8  (9.7-23.9) | 5.6  (0.6-23.1) | 30.9  (21.6-41.5) | 28.6  (18.6-40.5) | 38.9  (19.4-61.7) | 39.9  (39.3-40.4) |
| 3-5  (95% CI) | 14.3  (8.9-21.4) | 13.9  (8.2-21.6) | 16.7  (4.9-38.1) | 17.3  (10.3-26.6) | 19.0  (10.9-30.0) | 11.1  (2.4-31.1) | 28.2  (27.7-28.8) |
| Anxiety/Depression  1  (95% CI) | 63.9  (55.0-72.1) | 63.4  (53.7-72.3) | 66.7  (43.7-84.7) | 70.4  (59.8-79.5) | 73.0  (61.2-82.8) | 61.1  (38.3-80.6) | 62.8  (62.2-63.4) |
| 2  (95% CI) | 26.9  (19.5-35.3) | 29.7  (21.5-39.1) | 11.1  (2.4-31.1) | 23.5  (15.3-33.5) | 20.6  (12.1-31.8) | 33.3  (15.3-56.3) | 28.8  (28.3-29.4) |
| 3-5  (95% CI) | 9.2  (5.0-15.4) | 6.9  (3.2-13-1) | 22.2  (8.0-44.6) | 6.2  (2.4-13.0) | 6.3  (2.2-14-4) | 5.6  (0.6-23.2) | 8.4  (8.0-8.7) |

**Supplementary material.** The EQ-5D-5L results and the Swedish characteristics. All dimension levels are: 1 = no problems, 2 = slight problems, 3 = moderate problems, 4 = severe problems, 5 = unable/extreme problems

^1^Burström et al (16)
